# Gender discrimination in the emergency services: Female paramedic experiences in South Africa

**DOI:** 10.4102/phcfm.v17i1.4945

**Published:** 2025-09-03

**Authors:** Andrew W. Makkink, Busisiwe E. Nkhoma

**Affiliations:** 1 Department of Emergency Medical Care, Faculty of Health Sciences, University of Johannesburg, Johannesburg, South Africa

**Keywords:** gender discrimination, emergency medical services, paramedic, gender bias, patriarchy

## Abstract

**Background:**

Gender discrimination (GD), particularly that against women, remains a challenge in the workplace and paramedicine is no exception. Discrimination against women persists despite, in many cases, their being more qualified than their male counterparts.

**Aim:**

The aim of this study was to explore GD in paramedicine using the perceptions and experiences of South African female emergency care practitioners (ECPs).

**Setting:**

The study setting was within the Johannesburg area in South Africa, and targeted female ECPs.

**Methods:**

This study used a qualitative description design to gather data using online or face-to-face interviews from seven participants. Interviews were transcribed verbatim, read vertically and horizontally, coded using ATLAS.ti version 22 software and analysed for categories and themes.

**Results:**

There were six dominant themes that emerged from the data: (1) GD remains prevalent in emergency medical services (EMS); (2) female ECPs were undermined in the workplace; (3) there were race factors related to GD; (4) gender stereotypes were based on physical capabilities; (5) the negative effects of GD in the workplace; and (6) maternal wall bias.

**Conclusion:**

Gender discrimination against women in EMS persists with females being stereotyped, undermined and subject to maternal wall bias. Effects of GD on participants included psychological stress, feelings of inadequacy, isolation, sadness and self-doubt.

**Contribution:**

There is a paucity of research on GD in African EMS. The findings of this study provide valuable insights into GD in the EMS workplace and contribute to the growing body of knowledge related to GD worldwide.

## Introduction

Gender discrimination (GD) is a form of inequality and unjust treatment based on gender, and remains an issue worldwide; particularly for women.^1^ Discrimination against women is an ongoing topic of focus around the world, but it continues to be prevalent in many workplaces, including within the paramedical domain.^2^ Women face GD despite having the relevant qualifications to excel and further themselves in their career, and their growth is often restricted by the attitudes or biases of many people in their workplaces.^3,4^ This is particularly true within paramedicine in African contexts, although there is limited research on the topic, and often contributes to a ‘glass ceiling’ — a term coined for the seemingly impenetrable wall that stands in the way of career progression and promotion.^3^ This study seeks to address the knowledge gap by focusing on a highly qualified and underrepresented group in Johannesburg, South Africa.

It is still common in society to openly label professions, such as fire, police, emergency medical services (EMS) or nursing as being male- or female-dominated, a major contributing factor to GD and the glass ceiling and its narrative.^4^ This is evident where despite women being highly qualified, they were less likely to practice at more advanced levels and remained underrepresented in leadership roles meaning that paramedicine still suffers from gender non-traditionality.^4^ There are several contributing factors to GD in the healthcare workplace including the historicity of male-dominance, discriminatory promotion practices, perceptions related to work-life balance and embedded cultural and patriarchal practices.^2,5^

In South Africa, the historical prehospital qualification matrix was one grounded in vocational qualifications, which ranged in duration from four weeks to nine months. This system was discontinued in favour of a National Qualification Framework-aligned three-tiered system with a one year higher certificate, two year diploma, and a four year honours-level, professional bachelor’s degree in health sciences (BHS).^6^ Each of the qualifications, whether vocational or tertiary, allows a person to register with the Health Professions Council of South Africa (HPCSA) who determine the capabilities and scopes of practice of prehospital emergency care providers.^6,7,8^ The graduate emergency care practitioner (ECP) registers with the HPCSA on the ECP register, which is the highest capability-based qualification in prehospital emergency care,^6^ and therefore, is the person who is ultimately responsible for patient care and management, thus fulfilling the team and system leadership role.

Underrepresentation is a significant contributing factor to GD.^9^ This is exacerbated by the ongoing exodus of South African ECPs to the overseas market, resulting in a decrease in total number and thus visibility of female ECPs within the EMS population nationally.^10,11,12^ This exodus means that the already small population of highly qualified ECPs is further limited and, the proportion of the total EMS population who are female ECPs becomes even smaller.^10,11,12^ This exacerbates the underrepresentation of female ECPs within the paramedical workforce.

Patriarchy is a system in which men hold power and women are largely excluded. It is a bias that results in limited access for women being presented opportunities in the workplace, reinforces the view that women are less capable and perpetuates occupational sexism.^13^ This can negatively impact women’s self-esteem, morale and productivity.^14^ Tuohy has described how discrimination, bullying and harassment cause unnecessary psychological stress and the knock-on effect on the individual’s workday and on society as a whole.^15^ A further bias, often linked to patriarchy, is that of maternal wall bias that relates to when motherhood factors into the bias women experience at work and can be linked to negative competence and commitment assumptions in the workplace.^16^ Patriarchal biases have negative effects on opportunities, emotional factors and the general perceptions of women in the workplace.

Discrimination because of physical capabilities refers to discrimination linked to a person’s muscular strength abilities and potentially ignores a person’s intellectual ability or ability to do the job, focusing rather on physical aspects of the person.^17,18^ This discrimination is not only evident in male colleagues, but patients can also be distrustful of female emergency personnel’s ability.^19^ The association of physical prowess with competence is a potential barrier to a woman being recognised as workplace or clinical equals. Comments by colleagues relating to women’s lifting and strength limitations in EMS contribute to dropout because of ostracism, verbal and sexual harassment, social alienation and aggressive supervision.^20^ This suggests that the primary barriers women confront are likely to be social interactions related to the job, rather than professional requirements.

Despite protective legislation in South Africa, pregnancy remains a source of GD within the workplace.^21^ Pregnancy contributes to workplace discrimination against women by treating a woman (an applicant or employee) unfavourably because of pregnancy, childbirth or a medical condition related to pregnancy or childbirth.^21,22^ This ‘maternal wall bias’ or ‘maybe baby effect’ exacerbates GD beyond simply gender or physical ability in the workplace.^23^ Race is another contributing element to GD, and this is when someone (an applicant or employee) is treated unfairly because of their race and skin colour or complexion.^24,25^ Black women or women of colour, in particular, are susceptible to ‘double discrimination’, or discrimination based on both race and gender, as described by critical race theory.^26^ Being female, may still be a disadvantage in the paramedical workplace, compounded by pregnancy and racial differences to colleagues.

Despite policy interventions, legislation and employer narrative, GD remains a significant problem in the paramedical workplace and beyond. Furthermore, the effects of GD result in a greater susceptibility to developing anxiety disorders and depression with a potential for alcohol or drug abuse and even suicide.^27,28^ The aim of this study was to explore GD in prehospital emergency care using the perceptions and experiences of South African female ECPs.

## Research methods and design

### Study design

This exploratory, descriptive qualitative study sought to understand the perceptions and experiences of female ECPs relating to GD in the prehospital emergency care workplace. This design was used to facilitate a deep understanding of participants’ experiences and complex realities within their own specific contexts. Data were collected between July and August 2022.

### Researcher characteristics and reflexivity

The principal researcher (BN) was an African female, final year student studying for an honours-level degree in Emergency Medical Care in Johannesburg, South Africa. She conducted the interviews and, as an African female, had her own biases regarding the topic being investigated, thus there were ongoing engagements within the research team to mitigate this. The supervisor (AM) was a male academic with a PhD in Emergency Medicine and the expertise in quantitative, qualitative and mixed-methods research. The differing backgrounds and experiences of both researchers related to GD helped to balance their interpretation and both researchers were constantly aware of their own potential biases and maintained reflexivity throughout the study where they regularly reflected on and discussed their potential personal assumptions, biases and other influences that may have affected the research and its findings.

### Setting

The study setting was within the Johannesburg area in South Africa and targeted female ECPs. The setting was chosen as Johannesburg is one of the most populous cities within South Africa and being an urban area with a university where ECPs are trained, there was a higher concentration of potential participants. Emergency services in South Africa face several challenges that include high rates of violence and trauma, under resourcing, long response and transport times, and growing concerns regarding practitioner safety. Paramedical services are provided by state- and private-funded organisations and often interact with the fire service or other services not directly involved in patient care. Patriarchy is a historical problem in South African emergency services that seems to now manifest within paramedicine and affects persons working in this domain of the healthcare system.

### Study population and sampling strategy

The study population was female ECPs working within the Johannesburg area. Female ECPs from both state- and private-funded EMS were approached to participate in the study. There were no restrictions placed on age, working experience or role. The limited population of ECPs meant that potential participants who worked within the study area were purposely identified and approached within the study area to make up the sample. Purposive sampling was used to identify potential participants who were contacted on WhatsApp, and the research information was provided to them. Potential participants were sourced from institutional and work-based WhatsApp groups that the principal investigator (PI) and supervisor had access to. Some participants, upon hearing of the project, directly contacted the PI for participation. All persons who indicated a willingness to be involved were included in the sample. The shortage of female ECPs within the study area contributed to the limited sample size. The sample size was determined using the principles of data saturation. Coding and analysis were carried out after each interview and after seven interviews, data saturation was deemed to have been reached. This was because there were no new codes or themes emerging and the meaning of the existing codes relating to GD within paramedicine were repeating without further explanation needed.^29^

### Data collection

The research team developed an interview guide (Appendix 1) through a desktop study of recently published articles and refined this through discussion. The interview guide began by asking the participant demographic questions, explored their experiences of GD in the workplace with examples, explored contributing factors and finally the effects of GD on their professional and personal lives. The interview guide allowed for flexibility and probing questioning as well as for participants to express themselves freely. Upon indicating a willingness to participate, an arrangement was made to meet either physically or online at a time and place convenient to both the PI (BN) and the interviewee. All interviews were conducted by BN in English and varied in duration between 9 and 23 min. The Zoom platform was chosen as the alternative to face-to-face interviews because it complemented and extended qualitative researchers’ options of generating rich data.^30^ Interviews were audio-recorded using two devices and were transcribed verbatim.

### Data analysis

A qualitative descriptive approach guided the data analysis.^31^ Interviews were relistened to and read both vertically and horizontally to ensure immersion. Verbatim transcriptions were imported into computer-assisted qualitative data analysis software (CAQDAS) ATLAS.ti (version 22.2.5.0, Scientific Software Development GmbH, Berlin, Germany) for data management and analysis. Coding processes were primarily inductive and followed an open coding strategy that focused on participants’ descriptions of GD in the workplace. Codes were grouped into categories that were further grouped into themes. First-pass coding was conducted by BN, and an independent recode was performed by AM. Coding trees were compared and where incongruency was observed, this was resolved by discussion and agreement on the most appropriate code, category or theme.

### Trustworthiness

Trustworthiness was enhanced by employing strategies related to credibility, dependability, confirmability and transferability. Credibility was ensured by using a code-recode strategy and the generation of thick, rich verbatim quotes. Dependability was ensured by using the code-recode strategy as well as keeping a detailed audit trail. Confirmability was ensured by constant reflection and reflexivity that was previously discussed as well as through the use of quotes in the voices of the participants. Transferability was enabled by providing detailed information on the setting and participants.

### Ethical considerations

Ethical clearance to conduct this study was obtained from the University of Johannesburg Faculty of Health Sciences Research Ethics Committee (No. REC-1581-2022). All participants provided signed consent to be interviewed and audio recorded. From the first contact, potential participants were made aware of the voluntary and confidential nature of the study and that they were able to withdraw at any time without consequence. Interviews were coded so that participants could not be identified, maintaining confidentiality and transcripts were de-identified where this was required.

## Results

A total of seven female ECPs participated in this study. Participants were able to choose their mode of interview and five participants were interviewed on a cloud-based video conferencing platform on Zoom and two through a face-to-face semi-structured interview. Participant and interview characteristics are depicted in Table 1.

**TABLE 1:** Demographics of participants.

Participants	Years qualified as an ECP	Sector in the EMS	Job description	Race or ethnicity
Participant 1	5	Private	Operations	White
Participant 2	2	Private	Operations	White
Participant 3	2	State	Operations	Indian
Participant 4	3	Private	Operations	Black
Participant 5	3	State	Operations	Black
Participant 6	9	State	Managerial	Black
Participant 7	4	Private	Operations	White

ECP, emergency care practitioner; EMS, emergency medical service.

### Identified themes

There were six dominant themes that emerged from the data: (1) GD remains prevalent in the EMS; (2) female ECPs were undermined in the workplace; (3) racial and cultural contributors to gender discrimination; (4) gender stereotypes were based on physical capabilities; (5) negative impacts of gender discrimination in the workplace and coping mechanisms; and (6) maternal wall bias.

#### Theme 1: Gender discrimination remains prevalent in the emergency medical service

Participants indicated that GD remained prevalent in the EMS and identified several contributing factors. Identified sub-themes were that the EMS remained a male-dominated environment, that females were perceived as not being equal or inferior to their male counterparts and that the ability to do the job was based on physical capability.

Participants indicated a strong perception that the EMS was still a male-dominant environment and that males were associated with being in management positions or the highest qualified person:

‘And adding on top of that, being female, it makes it even worse because, remember our work in EMS, it is male-dominated.’ (Participant 6, managerial, black person)

‘… So I think there is that thing where, initially, you could call the whole paramedic field a man’s world; … So I think for a very long time it was male-dominated, it might still be male-dominated, I am not really quite sure how the stats are looking.’ (Participant 2, operations, white person)

‘So personally, my experience, especially in the area that I work in, people are so used to the male, or the guys, or the men being the ones who are in senior positions, so whether [*you’re a*] ECP or Critical Care Assistant (CCA), or whatever, but ALS paramedics usually, it’s men, you know, it’s the first time that they get a female ALS.’ (Participant 4, operations, black person)

There was a perception that male domination was related to the traditional male dominance of the prehospital emergency care profession and EMS workplace. Participants indicated that although this patriarchal narrative was still prevalent, it was seemingly changing over time:

‘I think historically, I think it’s a historic thing where the EMS was predominantly populated by males … So I think historically it is because it was a male-dominated industry, so only now are we having females coming on board.’ (Participant 5, operations, black person)

‘I think it is the general patriarchal society, where men have been dominant in many fields, in working, and are always seen as the ones in charge, and historically have been placed in positions of leadership and positions of power … I do think it is changing as the years go on, it is definitely easier now than it was, for argument’s sake, ten or twenty years ago, can you imagine?’ (Participant 1, operations, white person)

Interestingly, one of the participants highlighted that female representation within the EMS was improving and that this resulted in decreased discrimination because of a more prominent voice of females within the EMS:

‘… I feel there’s a lot more representation from women in the last couple of years whereas when I was a student, you wouldn’t find a lot of females. You would mostly just be working with men and the crew’s men and everyone’s men. Now I feel like there’s quite a nice distribution. It is still predominantly male-dominated, but I feel we’re getting a lot better and the more women that join, the less discrimination I feel happens because there’s a bigger voice.’ (Participant 7, operations, white person)

The narrative went further where participants indicated that there was a perception in the EMS that, as females, they were not equal to or were inferior to their male counterparts because they were female. This resulted in a general perception that females would overreact and there was a lack of respect with females being underestimated because of their gender:

‘Whereas a male, usually, naturally, they just get that respect from people, they get that recognition from people. As opposed to, with the female, a lot of people would want to maybe take advantage of you, or step on your head, or things like that, because you are a female … So, like I say, it is just that they underestimate you, it’s like they take it that because you are a female, you probably don’t know as much as the males that they are used to.’ (Participant 4, operations, black person)

There was a perception that the ability to do the job was based on traditionally male traits and the associated ability to deal with certain situations. This was also prevalent in the stereotypical perception that only males could be courageous, brave or strong enough to work within the EMS:

‘Maybe you go to a specific scene, they would inform you that maybe the scene is unsafe, they think the one thing that [*deleted name*] would do in an unsafe scene is just scream, because I am a female, I won’t be able to stand up and say; “You, run, do this.” Because females, what do they do in danger? They just scream.’ (Participant 6, managerial, black person)

‘… you get people who believe that in such an industry as ours, females aren’t allowed to do certain things, or females cannot do certain things, it is the male who’s maybe courageous enough, or is maybe brave enough, or who’s strong enough to do certain things, that’s just people’s beliefs. Some of them believe, in the workplace, that no, as a female you are not supposed to do this because it will be better for a man to do it.’ (Participant 4, operations, black person)

#### Theme 2: Female emergency care practitioners were undermined in the workplace

Despite being the highest qualified person in the workplace or on an incident, female ECPs indicated that there was still some narrative of undermining within the workplace. This was directly related to their gender and was perpetuated by healthcare professionals, patients or their family members. However, there were participants who indicated that this was becoming less pronounced:

‘… where working with a male counterpart or a male practitioner who is less qualified than me, significantly less qualified than me, and the patients, doctor, other health care providers, other people that we work [*with*] within the industry, rescue and fire, automatically assume that they are in charge, the male is in charge, where I am supposedly the person who is actually in charge, who has the qualification and can actually treat the patient. And based off nothing other than male versus female.’ (Participant 1, operations, white person)

‘I feel just in terms of maybe getting on a call and having everyone immediately look to the man and be like, he must be in charge because he’s the man and when you give your advice even though you’re the highest qualified, they want to know what does the man think. So, I think there’s still that element of almost passive discrimination.’ (Participant 7, operations, white person)

Interestingly, one participant mentioned that their being undermined or made to feel inferior was not always deliberate:

‘… so being dismissed just because I am a female, definitely is not a nice feeling. And often, the person who is making me feel inadequate or insufficient, doesn’t [*even*] realise what they are doing, and whether intentional or not, it still means that I am seen as not as good, or [*I*] feel less than.’ (Participant 1, operations, white person)

#### Theme 3: Racial and cultural contributors to gender discrimination

Participants also expressed how race or culture was exacerbating factor to GD in the prehospital emergency care domain:

‘… and they won’t think that of you, they will only think that of you if maybe you are another race, or maybe you are a male. But if you are black and you are a female, you are never seen to be in that specific hierarchy of EMS.’ (Participant 5, operations, black person)

‘… it is also cultural things, specifically in South Africa as a white female I am also discriminated against, for example, with black Zulu males, specifically, I struggled significantly to earn their respect and to get them to listen to me, because as a female, I am already seen as less than, in their culture, and now as a female who is in charge of them, and I happen to be white, you really struggle to get the respect earned, and for them to take you seriously …’ (Participant 1, operations, white person)

‘Even at times when you say your qualification, not even when you say your qualification, when they meet you and you say you are an ECP, they are like; ECP? And I am like; yes. It is like a shock to their system, that how can I be black and an ECP… because we are not seen as people who can go up the ladder, we can only stop at this level.’ (Participant 6, managerial, black person)

#### Theme 4: Gender stereotypes were based on physical capabilities

Participants highlighted how gender stereotypes were grounded in historicity and often based on physical capabilities, inadvertently perpetuated discriminatory ideas and further oppressed women in the workplace. The women in this study mentioned the preconception about the physical attributes or characteristics that were possessed or performed by women and men in the field. The participants revealed that physical capability-based gender stereotypes undervalued their capabilities:

‘I think it’s got to do more with people’s beliefs. I think whether it’s their personal beliefs or their professional beliefs, you get people who believe that in such an industry as ours, females aren’t allowed to do certain things, or females cannot do certain things, it is the male who’s maybe courageous enough, or is maybe brave enough, or who’s strong enough to do certain things, that’s just people’s beliefs. Some of them believe, in the workplace, that no, as a female you are not supposed to do this because it will be better for a man to do it.’ (Participant 4, operations, black person)

‘… those are some of the issues that, in the black community, we come across. And because of that, then you can be looked at as weak, because you are a woman, you don’t have a place in society to have a voice.’ (Participant 5, operations, black person)

‘I think the one thing, our career involves us driving, so you would be associated as somebody who would never be able to respond. Because already they are like, females, they will panic, the moment that car comes between her she will panic … I don’t have the guts to hold that steering wheel … They don’t think we are capable.’ (Participant 6, managerial, black person)

‘[*Y*]ou’ll get to a call and they go: “You can’t lift them, you’re a small woman, you won’t be able to lift, you can’t carry this or that”.’ (Participant 7, operations, white person)

#### Theme 5: Negative impacts of gender discrimination in the workplace and coping mechanisms

Participants highlighted how GD had a detrimental psychological impact that elicited negative emotions and had the potential to reduce their self-confidence. Participants indicated that they experienced several negative emotions because of GD in the workplace. These included feelings of isolation, sadness, anger, discouragement, disappointment and self-doubt. Within this, though, was an individual sense of pride and achievement of what it meant to become an ECP, which was undermined by the lack of acknowledgement of their qualification and skillset:

‘It is definitely a negative feeling. You don’t want to be seen, obviously, less than. And it makes you feel isolated, it undermines your worth. I have studied as long or as hard, if not longer and harder than other people to get [*to*] where I am, so being dismissed just because I am a female, definitely is not a nice feeling … There is obviously a feeling of disappointment, feeling of sadness that comes towards … But also, in the same way, feelings of anger.’ (Participant 1, operations, white person)

‘It definitely discourages us. Because the thing is, you try and be the best practitioner and a good person, not just your professional, but your personal side.’ (Participant 2, operations, white person)

‘It’s stressful and it’s frustrating, it makes me angry that we work so hard to obtain this degree, and we are there just to work, and just randomly get victimised or picked-on, or spoken to inappropriately, I think it is very unfair.’ (Participant 3, operations, Indian person)

‘There used to be a bit of self-doubt, I used to pre-question myself, that; okay, if I decide that this is what I want to do on a patient, are they going to respect it, because I am the one who said; no, let’s do this, or are they actually going to actually go with it? So there used to be a bit of self-doubt, it affected my self-confidence a bit. So that’s more professional.’ (Participant 4, operations, black person)

‘This has affected me, is if you’re perhaps in a challenging situation or you’re feeling like you aren’t entirely confident in your decision making at that time that can be made worse when you feel you’re up against having to prove yourself. Or that person already doesn’t trust or believe in me; I’m struggling to trust or believe in myself; it makes it worse but as a whole.’ (Participant 7, operations, white person)

One participant highlighted how GD was also perpetuated by women and that being an authentic woman was discouraged:

‘… a lot of the discrimination from other women in the field, but that are, can I say, old school. They came from the time when they entered the male-dominated field and had to act like one of the boys, type of thing. So then if you enter the field, and you’re like; I am going to be authentically a woman; and I am not scared to say it, I am not going to try and be a man, then they are; oh, you are just flaunting your sexuality. That has nothing to do with it, but it is like there is this misnomer of how you are supposed to act in that area.’ (Participant 2, operations, white person)

Participants indicated that they coped with GD by separating their personal lives from their work lives, ignoring the issue or by engaging in physical activity. Primarily, although, participants indicated that they dealt with the issues related to GD on their own or used family members for support:

‘… I usually draw the line between my workspace and my personal life. I don’t let the two intertwine with one another, so whatever happens at work, I try my best to sort it out at work. If I need maybe a family or a friend to support me … then I do have a support system in my personal life that can help me through everything.’ (Participant 4, operations, black person)

‘So as Paramedics we very good at isolating incidents and putting things in a box on the shelf. So you learn to take it in your stride, you learn to ignore the comments, you learn to ignore the chirps … And you get used to ignoring it, you get used to brushing it off, and very rarely confronting the issue, unless it is not always productive to have conflict, especially when on a scene where you are trying to treat a patient, it is not always productive to be confrontational, because then who suffers? … And I guess every now and then you do take it home with you and you think about [*it*] and reflect on it, but most of the time the issues lies with the person, not with me.’ (Participant 1, operations, white person)

‘But that is one of the coping mechanisms, which is avoiding the problem, there. And then coming home, nowadays when I avoid, I don’t have that stress, so in general, stress management is usually managed through workouts, I work out at a gym all the time. I burden off there, I listen to music. I really do have my own space. I do my own thing. Sometimes I vent to my boyfriend and my mom, sometimes. But yes, the venting does help, as well.’ (Participant 3, operations, Indian person)

#### Theme 6: Maternal wall bias

Maternal wall bias was a strongly grounded theme, and participants indicated that they felt discriminated against because of the actual or potential maternal role that they were or would be performing. Maternal wall bias was also linked to the biological role that a mother played in raising a child:

‘I think maybe it could, but I feel like it shouldn’t, because the moment it does, I feel like we are discriminated against for playing our natural duties of which eventually you are going to be a mom or a wife. But I do feel like it affects you to [*an*] extent, because I know that the year I started working, I fell pregnant, so the following year, I gave birth, and then I wasn’t at work for five months. So automatically, apparently that prolongs your probation … So I think this one would apply to every female in whatever role they play at work, whether you are [*in*] leadership or you are just [*part of*] the crew … It affects all the women across all boards, across every race; it is just there. So what I am trying to say is; that as much as I feel like society has made it to hamper our duties at work, but it shouldn’t. [*W*]e shouldn’t be discriminated against, I shouldn’t be afraid to have my third child three years in a row, because at work I am going to lose out on this or lose out on that. But in a sense, it does take away from what we eventually become.’ (Participant 5, operations, black person)

‘No, I don’t think so. Because me being a mother, is the same as another EMS guy being a father. The only thing that makes it difficult for us, is that when are pregnant and you are a mother within EMS, things become different for you, because you need to go back to your shift within four months, and therefore you are breastfeeding, and in the environment that you are in, it is not easy.’ (Participant 6, managerial, black person)

## Discussion

The women in this study reported experiencing discrimination from a variety of perspectives and consistent with the literature, our data revealed that discrimination in the workplace experienced by females remains common.^19^ Participants identified several negative consequences of GD with corresponding detrimental impacts on mental health.

Participants in this study highlighted that the perception remains that EMS is a male-dominated industry, and that females are underrepresented in the workforce. Even though more women are pursuing the profession, females were still perceived to be a minority. Although not part of the initial aim, there was a perception that women were underrepresented in the workplace, especially in management and leadership roles. Several studies have also been linked to the fact that women remain underrepresented and disrespected at every level in other countries even though they have successfully earned higher qualifications than males.^32,33,34^ Several participants in this study perceived themselves as being both underrepresented and not adequately respected despite being highly qualified and highly competent.

Despite more companies committing to gender balance, progress is still slow, especially when it comes to women pursuing careers that are dominated by males.^10,32^ In workplaces where men dominate, such as the EMS, there are disproportionately few opportunities and little representation of women, which may contribute to GD.^3^ Evidence persists that women have been and remain underrepresented, subjected to discrimination and disregarded when it comes to leadership roles in the workplace.^10,33,35^ These findings were confirmed by participants in this study who highlighted that underrepresentation of females within management structures remained a concern. Although the reasons for female underrepresentation may be varied, the fact that they persist is a point of concern and should be further explored.

The participants mentioned that the presence of women in the EMS has improved significantly since the past, with a more favourable distribution, despite the fact that it is still dominated by males. They believe that if there is more female representation, it would result in less discrimination. Emergency medical services have traditionally been dominated by men, which naturally led to the majority of leaders in such professions being men.^10,33^ However, there is a modest but steady balancing of the gender ratio within the profession.^36^ Participants indicated that an increasing female workforce had the potential for several positive outcomes.

The participants in this study highlighted that undermining discriminatory practices originated in the patriarchal system and strongly favoured men over women, perpetuating oppressive gender norms for women in the workplace. Studies have demonstrated that in patriarchal societies, women encounter gender-power relations that directly contribute to women having a lower role in society and in the workplace; and that in patriarchal societies, males are viewed as superior to women and their responsibilities are defined in the workplace, while women are restricted to the domestic sphere and have nothing to contribute outside of the house.^37,38,39,40,41^ The women in this study worked in predominately male environments where GD and patriarchal norms were strongly ingrained.

Racism in the EMS is often described from the perspective of patient care and allocation of resources, and treatment bias. Participants in this study highlighted how racism affected how they were treated and recognised by colleagues, but there appears to be a paucity of literature related to direct collegial racism within the EMS. Racial discrimination has been shown to negatively affect the mental health of health care workers highlighting the importance of addressing this behaviour.^42^ Racism extends into health care education and the impact of racial bias in health professions education as well as the long-term impact of awareness and knowledge of racial bias have been recognised as areas requiring further investigation.^43^ Given that GD and racism seem to sometimes go hand-in-hand, appropriate education programmes may help mitigate both GD and racism within the EMS. In addition, strategies to deal with the effects of these should be explored.

Previous studies have shown that gender stereotypes and the nature of the job and its perceived effect on work-life balance may play a role in discrimination, however, it is unclear how these components interact to promote GD in everyday workplaces.^32^ Gender discrimination can manifest in work-life balance challenges driven by the patriarchal biases within society and are more intense in developing countries (such as South Africa).^41^ This was prevalent in the narrative of the participants related to their ability to be a mother and a paramedic. The stereotypical view of females expressed in this study also involved the idea that women were perceived to lack necessary capabilities, such as physical abilities, and that they were unable to perform typical activities that men were considered capable of performing, which include navigating their personal response vehicle, carrying equipment and lifting capability. Participants indicated that they were stereotypically viewed as weak or incapable and also mistaken for someone that was in a more junior position, reinforcing the perception of gender stereotyping still being prevalent.

The effects of GD on female employees are varied and far-reaching. Published evidence links females’ lack of confidence in the workplace, similar to that voiced in this study, appeared to have a negative impact on their career progression.^44^ Despite their high qualifications women in literature, like those in this study, often expressed doubts about their own ability and competence in the profession.^19,44^ The women who participated in this study mentioned that they were sometimes treated as if they were inferior in the workplace, especially when they felt the need to constantly had to prove their skills and capabilities.

Some effects of GD resulted in participants employing coping and other strategies to deal with the stress. Coping refers to the strategies that people use to deal with a situation or regulate their emotions to maintain their well-being.^45^ Some strategies described by participants included avoidance of the issue and internalising or accepting the discrimination. Some of the participants mentioned that they had become accustomed and that they ignored the remarks and comments. As women in this study encountered greater GD and victimisation, they engaged in a range of coping strategies to manage the stressors that came with it. Some attended gym to do workouts, isolated themselves from the situation or engaged with family members. A lack of social support was also an identified factor exacerbating the effects of GD. This corresponds with literature where there is limited social support and, as a result, a greater risk of adverse effects related to GD.^19,20,43^ The participants suggested that social support can mitigate the effects of GD, and some women have a support system at home and colleagues at work that they can vent to. In line with previous research, it was discovered that professionals who had strong spousal and friend support against GD reported better mental health. Social support, in general, has been demonstrated to help people deal with stressors caused by discrimination.^46^

Gender discrimination remains an issue within the EMS and evidence suggests that progress, while slow is taking place.^10^ However, participants in this study suggest that there is an encouraging evolution underway. This study highlights some of the ongoing issues experienced by female ECPs within the Johannesburg area and provides a valuable contribution to the existing body of knowledge related to GD in the EMS.

### Strengths and limitations of the study

The care taken to ensure trustworthiness was a key strength of this study, and the use of the generic qualitative description design with thick, rich quotes allowed the voices of the participants to come through strongly. As with all qualitative research, data interpretation involves a degree of subjectivity; however, this was addressed through reflexivity, a code-recode process, and collaborative theme development to enhance trustworthiness and transparency.

Limitations of the study include those commonly linked to using a generic qualitative descriptive design, such as social desirability bias where participants may have provided a narrative that they believed was what the researchers were expecting. Although the research involved seven participants, given the low numbers of female ECPs within the study area, this may not be a significant limiting transferability factor. However, the use of only one qualification and gender group from one geographically limited area limits the transferability of the results to other qualifications and therefore the EMS and paramedicine as a whole. That said, the results do provide a window into GD in the EMS and serve as a valuable contribution to the body of knowledge. The quantity of data may have resulted in coding fatigue, but the independent code-recode strategy helped to alleviate this. It was interesting to note that the interviews were relatively brief given the topic under investigation. We did not explore this during data collection but postulate that the sensitivity of the topic and the emotions that participants may have felt describing their experiences could be reasons.

### Implications or recommendations

The recommendations were made within the context of GD within the paramedicine operational, educational and research environments.

It is recommended that paramedicine as a profession adopts a stronger stance on GD and racial bias within the workplace. All healthcare professionals involved in the prehospital space should undergo gender sensitivity training to better understand GD and racial bias and their implications both in the profession and to persons affected by GD. Educational institutions should include aspects of GD and racial bias into their curricula to ensure that students are not only adequately prepared for potential GD in the workplace but are also cognisant of their own biases. Industry role-players should take meaningful steps to addressing gender imbalances within the workforce and should advocate for equality of staff regardless of perceived physical prowess of genders.

The effects of GD and race bias are profound and support mechanisms should be put in place to support victims of these behaviours. It is also important that perpetrators of GD and racial bias are identified and counselled appropriately to ensure that their behaviours are not perpetuated. The importance of support from industry, healthcare professionals and support staff should be highlighted and encouraged in order to build a more inclusive workplace.

The paucity of local research and the constantly evolving nature of GD in the EMS highlights the need for, and importance of, further research. As a result, future research should include a wider range of qualifications and should focus on a wider geographical area. It is also important to explore the male perspective on GD within the workplace, and future studies should also include male participants and include a focus on discrimination against other marginalised groups.

## Conclusion

This study identified several issues that persist relating to workplace discrimination against women in the EMS. Despite a global narrative to improve working conditions for women, various patterns of gender biases and discrimination against women in the workplace persist. The study identified six dominant themes related to GD within the EMS: gender discrimination remains prevalent in the EMS, female ECPs were undermined in the workplace, there were race factors related to GD, gender stereotypes were based on physical capabilities, the negative effects of GD in the workplace and maternal wall bias. There were also various significant negative effects of GD, such as psychological stressors, limited career progression or advancement limitations and the creation of feelings of inadequacy or lack of confidence in themselves. Negative emotions such as isolation, sadness, anger, discouragement, disappointment and self-doubt were directly linked to GD. These findings highlight the urgent need for gender-transformative policies within EMS and reinforce the value of inclusive leadership practices.

## Appendix 1: Interview guide

1. How long have you been qualified as an ECP?
2. How would you describe your current role (management, operations, administration)?
3. What is your experience of gender discrimination in the emergency medical services?
4. Please provide some examples of your experiences of gender discrimination in the workplace?
5. What factors do you believe contribute to gender discrimination in the workplace?
6. What effects has gender discrimination had on your professional and personal life?
